# Cell-Based Regenerative Therapy as an Alternative to Liver Transplantation for End-Stage Liver Disease: Experience from Iran

**Published:** 2010-02-01

**Authors:** R. Malekzadeh, M. Mohamadnejad, K. Alimoghaddam, M. Bagheri, H. Baharvand, A. Ghavamzadeh

**Affiliations:** 1 *Digestive Disease Research Center, Tehran University of Medical Sciences, Tehran, Iran. *; 2 *Hematology, Oncology, BMT Research Center, Tehran University of Medical Sciences, Tehran, Iran. *; 3 *Department of Stem Cells and Developmental Biology, and *; 4 *Department of Regenerative Medicine, Royan Institute for Stem Cell Biology and Technology, ACECR, Tehran, Iran.*

**Keywords:** Stem cell, liver transplant, cirrhosis, cell therapy, bone marrow

## Abstract

Several types of cells including mature hepatocytes, adult liver progenitor cells and human embryonic stem cells, fetal liver progenitor cells, bone marrow derived hematopoietic or mesenchymal stem cells, and umbilical cord blood cells—both in rodents and humans—have been reported to be capable of self-replication, giving rise to daughter hepatocytes, both *in vivo* and *in vitro*. They have been shown to be able to repopulate liver in both animal models of liver injury and in patients with liver disease and to improve liver function. Human embryonic stem cell therapy seems to be a great promise for the treatment of liver cirrhosis, but there is no human clinical application due to ethical concerns or difficulties in harvesting or safely and efficiently expanding sufficient quantities. In contrast, adult bone marrow-derived hematopoietic or mesenchymal stem cells, which can be easily and safely harvested, have been used in clinical trials to treat several chronic diseases including chronic liver disease. Cell therapy offers exciting promise for future treatment of cirrhosis and metabolic liver diseases, but significant technical hurdles remain that will only be overcome through years of intensive research. There is also serious concern about the long-term safety of stem cell therapy and the possibility of tumor development. Herein, we present our experience with cell therapy in treatment of chronic liver disease in Iran.

## INTRODUCTION

Liver fibrosis represents the common endpoint of the majority of chronic liver injuries [1]. Cirrhosis represents a late stage of progressive hepatic fibrosis characterized by distortion of the hepatic architecture and the formation of regenerative nodules [2]. 

Patients with cirrhosis are susceptible to a variety of complications and their life expectancy is markedly reduced. Reducing liver fibrosis or delaying its progression may result in longer patient survival and thereby, a reduced need for liver transplantation [3]. While successful treatment of the underlying cause may lead to regression of liver fibrosis [3, 4], or even regression of early stages of cirrhosis [1, 2], this is an occasional finding and removing the underlying cause is not possible in many patients. A reliable way and an effective strategy to reduce liver fibrosis have still not been reported. The standard treatment for decompensated cirrhosis is liver transplantation. However, it has several limitations, including, scarce organ resource, resulting in sizable mortality among patients on the transplant waiting list, cost of surgery and hospitalization, follow-up and life-long immunosuppressive drug therapy with considerable long-term side effects.

Herein, we report on our experiences on stem cell transplantation in human liver cirrhosis. The objective of our studies was to evaluate the safety and the feasibility of autologous stem cell transplantation in decompensated cirrhosis.

**STEM CELLS AND TYPES**

Most cells in the body, such as heart or skin cells, are committed to conduct a specific function. Stem cells, however, are uncommitted cells that have the ability to self renew and differentiate into a functional cell type [5]. Conventionally, stem cells are either classified as those derived from embryo or adult tissues. Embryonic stem cells (ESCs), derived from the inner cell mass of blastocysts, are pluripotent and have the ability to entirely colonize an organism and give rise to almost all cell types [5]. Stem cells found in adult organisms are referred to as “adult stem cells,” and are present in most, if not all, adult organs [5]. They are considered multipotent, since they can evolve into mature cell types of one or more lineages, but cannot reconstitute the organism as a whole. What determines stem cell potency depends to a large extent on the genetic make-up of the cell and whether it contains the appropriate genetic circuitry to differentiate to a specific cell type. However, the decision to differentiate or self-renew is often regulated by the stem cell microenvironment, also known as the “stem cell niche.” For example, changes in cytokine gradients, cell-cell and cell-matrix contacts are important in switching “on” and “off” genes and gene pathways, thereby controlling the type of cell(s) that are generated [5].

**EMBRYONIC STEM CELLS**

Human ESCs were established by Thomson, *et al* [6]. ESCs have been found to have potentials to be precursors for organ specific cell lines including hepatocytes. But due to ethical concerns, the United States FDA has just recently (January 2009) approved the first human clinical trial of cells derived from human embryonic stem cells. Therefore, research using these cells in clinic is still in its early stages and that is why we still do not know whether tissues derived from embryonic stem cells would cause transplant rejection or not [7].

**ADULT STEM CELLS**

The ability of adult tissues such as skin, hemopoietic system, bone, and liver to repair or renew indicates the presence of stem or progenitor cells. The use of autologous or allogeneic cells taken from adult patients might provide a less difficult route to regenerative-cell therapies. In adults, stem cells are generally thought to be tissue-specific and are able to be lineage restricted and therefore can only differentiate into cell types of the tissue of origin [5]. However, several recent studies suggest that these cells might be able to break the barriers of germ layer commitment and differentiate *in vitro* and/or *in vivo* into cells of different tissues. For example, when bone marrow is extracted and the cells are placed in a plastic dish, the populations of cells that float are blood-forming stem cells—hemopoietic stem cells (HSCs)—and those that adhere to the dish are referred to as “stromal cells” including mesenchymal stem cells (MSCs). These cells can replicate as undifferentiated forms and have the potential to differentiate into lineages of mesodermal tissues including bone, cartilage, fat, muscle and hepatocytes [8, 9]. Moreover, transplanted bone marrow cells contribute to endothelium and skeletal muscle myoblasts and acquire properties of hepatic and biliary duct cells, lung, gut, and skin epithelia as well as neuroectodermal cells [9, 10]. Jiang and coworkers, recently demonstrated a rare multipotent adult progenitor cell within MSC cultures from rodent bone marrow which could differentiate not only into mesenchymal lineage cells but also into endothelium and endoderm [11]. 

**BONE MARROW-DERIVED STEM CELL TRANSPLANTATION FOR LIVER DISEASE**

It has been shown that during tissue injury or inflammation, bone marrow stem cells migrate to the injured organ to maintain homeostasis [12]. This important theory has formed the basis for regenerative therapy whereby treatment with appropriate stem cells might be used to treat several specific diseases including chronic liver diseases [12]. Studies in rodent models of liver disease have confirmed that following hepatectomy, liver cells are able to undergo numerous cell divisions maintaining their fully differentiated state to compensate for hepatocyte loss and the undifferentiated liver progenitor cells; the resident hepatic stem cells play only a minor role in this process but after acute necrosis or chronic liver diseases such as viral hepatitis or alcoholic liver diseases, hepatocyte progenitor cells play an important role [7]. These cells which originate most likely from bone marrow, express markers of both hepatocyte lineages and the biliary epithelium and have also been shown to express HSC markers CD34, c-kit (CD117) [13] and Thy (CD90) [7]. Plasticity of bone marrow-derived stem cells (BMSC) has been suggested for a number of different tissue types and has generated hope of its use as a cellular therapy for a variety of diseases. This initial optimism has been tempered by a recognition that much of the observed plasticity occur at either a low level or is the consequence of cellular fusion rather than trans-differentiation [12, 13].

Adult stem cells and tissues derived from them are currently believed less likely to initiate rejection after transplantation. This is because a patient’s own cells could be expanded in culture, differentiated into a specific cell type like hepatocytes and infused into the same patient. This represents a significant advantage, because administration of immunosuppressive drugs, which should be used life-long after organ transplantation with huge cost and many side-effects, is no more necessary after cell therapy. Experience with use of blood-forming bone marrow stem cells, particularly autologous BMSCs, has unique advantages over other stem cell sources. The cells have been used very successfully during the past 40 years for bone marrow transplantation and advances in techniques of collecting and harvesting them have been already achieved. BMSCs have been used to reconstitute the immune system after leukemia, lymphoma or various blood or autoimmune disorders following immunosuppressive or chemotherapy. The non-malignant, non-hematologic clinical indications for use of BMSCs have also been demonstrated in the treatment of other chronic diseases such as diabetes [12]. Human ESCs can grow easily in culture while adult stem cell expansion is difficult and methods to increase their numbers in cell culture are more complex and challenging and this limitation is now the main barrier for adult stem cell replacement therapies since large numbers of cells are needed for effective therapy of chronic diseases such as cirrhosis [12]. The majority of human stem cell trials have used HSCs, MSCs, or both, which can be readily collected from bone marrow or peripheral blood using various methods like surface antigen detection by flow cytometry, clonogenic colony-forming assays, and *in vivo* transplant marrow repopulation assays [13]. 

During the last five years, several animal and human studies have demonstrated that both MSCs and HSCs could be used to treat liver cirrhosis [14, 15]. The basis for this therapeutic effect is the fact that diseased liver can recruit migratory stem cells from the bone marrow to generate hepatocyte-like cells either by cell fusion or trans-differentiation. These studies have shown hat bone marrow stem cell transplantation can lead to regression of liver fibrosis. 

**STUDY 1**

Our first pilot study [16] on feasibility of this method included four patients with decompensated cirrhosis who were on the liver transplant waiting list and received, via hepatic artery, a mean number of 5.25×10^6^ CD34^+^ (with 90.5% purity), subpopulation of autologous bone marrow-derived stem cells. These CD34^+^ stem cells were separated from almost 200 mL bone marrow aspirated from iliac crest. The study outcome included liver volume as assessed by computed tomography (CT), change in MELD score, and patient quality of life (Short Form-36) questionnaire. One of the patients died of hepatorenal syndrome shortly after the procedure and before liver transplantation could have been performed. Development of hepatorenal syndrome was thought to be due to contrast nephropathy [16]. The remaining three patients were followed over a six-month period and despite a trend towards increased serum albumin (from 30.7 to 33.7 g/dL) and a reduction in the prothrombin time (from 17.8 to 16.1 s) during this period, the mean MELD score increased from 16 at enrollment to 17. After the death of patient four, the study was terminated, prematurely. 

**STUDY 2**

Another pilot study [17] on feasibility of this technique was conducted investigating the effects of autologous bone marrow MSCs in four patients on the liver transplantation waiting list with decompensated cirrhosis. Bone marrow (80–100 mL) obtained from the posterior iliac crest was processed and cultured under appropriate conditions to isolate and expand MSCs. Each patient received a mean of 31.7×10^6^ cells infused into a peripheral vein over 30 minutes. One patient had autoimmune hepatitis and the three remaining patients had cryptogenic cirrhosis. Baseline characteristics were obtained at enrollment and monitored at regular intervals during 12 months of the study. The mean MELD at enrollment was 23 and decreased to 20 by the end of the study. Responses to the Short Form-36 questionnaire showed an improvement in quality of life of all patients (the mean physical component scale increased from 31.4 to 65.2, and the mean mental component scale increased from 36.3 to 65.6). Serial CT showed an increase in liver volume in three patients (mean value of 615 mL at baseline, and 866 mL six months after transplantation). We concluded that autologous MSC transplantation through a peripheral vein is safe and feasible in the treatment of liver cirrhosis. Improvements in liver function tests and MELD scores of some of our patients were promising, but this study is limited by the small number of patients enrolled and the lack of a control group in the trial. We also did not track the fate of the infused cells to clarify their putative mechanism of action [17]. 

**STUDY 3**

We subsequently designed the first randomized controlled study [18] evaluating the efficacy of autologous BM-MSC transplantation in patients with advanced chronic liver disease to determine the efficacy of MSC transplantation (compared to placebo group) and tracking the stem cells in the patient body. Thirty patients with decompensated cirrhosis were randomly assigned to either case or control groups. BM of patients in both groups was aspirated. In the case group, culture of MSC was done. The mean duration of culture was three months. In the control group, BM aspirates were cryopreserved, waiting for the results of the study. Similar to cases, after the presumed interval, infusion of 100 mL DW5 with vitamin B complex was done as placebo in the control group. Our plan was to culture and infuse the cryopreserved BM aspirates to control group if the results of the study was encouraging. Each patient was followed for one year. So far, we have included almost 30 patients in our study. The design, inclusion and exclusion criteria are as in phase 1 study, but because of increased facilities for the culture of MSCs, about 400×10^6^ cells were transplanted in each recipient. The patients were followed with laboratory tests, CT volumetric study and liver biopsy. In two patients, 10% of the cultured MSCs were labeled with ^111^In-Oxine and the cells were traced in the patients’ body by performing SPECT images. Immediately after intravenous infusion, the labeled MSCs first accumulated in the lungs, and gradually moved to the liver and spleen after several hours to a few days [19]. On SPECT images after 24 hours, the tracer distribution was homogenous throughout the liver and spleen. Region of interest (ROI) analysis in the first patient showed that the percentage of the homing of the cells into the liver (following decay and background corrections and geometric mean calculation) increased from 2.8% at the second hour to 13.5% by the 10^th^ day post-infusion. The percentages of cells in the liver of the second patient were 0% at the second hour and 13% after 10 days [19]. 

An independent group of hepatologists selected by DDRC research council would periodically review the results of interim analysis. The board is empowered to recommend termination of the study on the basis of safety concerns. An interim analysis for 12 patients and controls who had finished the one-year follow-up revealed that there is a numerical but not statistically significant trend for the improvement of albumin, prothrombin time, INR, total bilirubin, and platelets in the MSC group compared to the placebo group. The improvement was mild and more prominent after four months (0.8 mg/dL improvement in total bilirubin; 0.4 g/dL improvement in albumin; 10^4^/µL improvement in platelet counts, and 0.2 unit improvement in INR in the MSC group after four months). Since we observed mild improvements in all of the above-mentioned parameters, it is possible that these improvements are real, although the number of cases for this interim analysis was still too low (n=12 in each group), and the improvements were not statistically significant [18].

## DISCUSSION

Our study did not show unequivocal benefits of stem cell therapy in ESLD, but it confirmed the safety of this procedure with some favorable effects on a short-term basis. At least, it may be promising in serving as a bridge to liver transplantation. Other clinical trials investigating the effect of adult BMSCs in patients with liver disease including the effects of the mobilization of bone marrow cells using granulocyte colony-stimulating factor (G-CSF) were mainly uncontrolled and studied only a small number of patients [12, 20]. There are several important unresolved issues regarding the stem cell transplantation in the treatment of cirrhosis. The major challenges are: identifying the stages of liver disease at which stem cells should be used—the best type of stem cells to be infused; the minimum effective number of the cells; and the best route of administration [21]. Our studies showed that autologous MSC transplantation through a peripheral vein is safe and feasible in patients with liver cirrhosis. Improvements in liver function tests and MELD scores of some of our patients are promising. However, further controlled trials with longer duration of follow-up should be performed to better clarify the safety and efficacy of this treatment modality. Our studies also highlighted the previously-established short-term safety of stem cell transplantation [12, 20]. There are of course some mild common side-effects including pain at the infusion site, low grade fever, nausea and rash. Major concerns are medium- to long-term adverse effects of this type of therapy including progressive liver fibrosis and the development of hepatocellular carcinoma [21]. There is an accumulating body of evidence concerning the malignant potential and the liver fibrogenic ability (hepatic stellate cells and myofibroblasts) of BMSCs, although the exact parent cells have not yet been identified [21]. This raises concerns regarding the long-term safety of stem cell infusions, particularly in decompensated cirrhotic patients with minimal hepatic reserve. Furthermore, it has been suggested that poorly differentiated hepatocellular carcinoma originates from hepatic oval cells and BMSCs. Indeed, the transformation of hepatic oval cells to hepatocellular carcinoma cells has been shown in animal models. Similarly, *in vitro* data suggest that MSCs may undergo malignant transformation after repeated *in vitro* culture. Whether these data from animal models could be applicable in human is not still clear. But this potential long-term risk should be considered [21]. 

The only type of malignancy that has been reported to develop following HSC transplantation in humans is donor type leukemia [22], but it mainly occurs with a cord blood source of stem cells which is less differentiated and potentially more prone to transformation than the adult bone marrow or peripheral blood stem cells [22]. 

According to a very recent study by our group [23], using complete blood count, platelet count, serum alpha-fetoprotein level, abdominal ultrasound, and abdominal CT at the baseline and for every six months up to the end of a mean±SD follow-up of 23.5±9.7 months in 15 patients (six men) with a mean±SD age of 45.1±14.9 years, who were treated by HSC or MSC therapy for liver cirrhosis, none of the patients developed evidence of leukemia nor liver tumor, nor other intra-abdominal tumors. Therefore, at least in short- and medium-term basis, the risk of the development of HCC was nil in our cases [23]. 

Recently, a case of donor-derived multifocal brain tumor was reported four years after repeated transplantations of fetal neural stem cells in a patient affected by the neurodegenerative hereditary disorder ataxia telangiectasia [24]. The occurrence of a donor origin brain tumor in this report is the first proof of the concerns raised about the long-term risks of malignancy in stem cell transplantation. This reminds us of the occurrence of leukemia resulting from viral integration leading to severe combined immunodeficiency syndrome (SCID-X1).

Last year, the International Society for Stem Cell Research formed an international task force of experts in stem cells and clinical research to define guidelines for development of safe and effective stem cell therapies for patients. These guidelines are now available and define a “roadmap” for basic and clinical researchers, outlining what needs to be accomplished to move stem cells from promising research to approved treatments for patients [25]. Clinicians should not over-promise the benefits of stem cell therapy or down-play its risks. Stem cell therapy, like all other therapeutic modalities, should be evidence-based with clear scientific rationale [25]. 
